# Synthesis, structure, and theoretical studies of a calcium complex of a unique dianion derived from 1-methyl­pyrrolidin-2-one

**DOI:** 10.1107/S205698902001628X

**Published:** 2021-01-01

**Authors:** Ray J. Butcher, Andrew P. Purdy, Paul A. Brown, Daniel Gunlycke

**Affiliations:** a Department of Chemistry, Howard University, 525 College Street NW, Washington DC 20059, USA; b Chemistry Division, Code 6123, Naval Research Laboratory, 4555 Overlook Av, SW, Washington DC 20375-5342, USA; c Chemistry Division, Code 6189, ASEE Postdoctoral Associate, Naval Research Laboratory, 4555 Overlook Av, SW, Washington DC 20375-5342, USA; d Chemistry Division, Code 6189, Naval Research Laboratory, 4555 Overlook Av, SW, Washington DC 20375-5342, USA

**Keywords:** crystal structure, (*E*)-1,1′-dimethyl-2,2′-dioxo-1,1′,2,2′-tetra­hydro-[3,3′-bipyrrolyl­idene]-5,5′-bis­(thiol­ate), dianion, calcium-*N*-methyl-2-pyrrolidine coordination, density functional theory

## Abstract

The title compound, *catena*-poly[[tetra­kis­(1-methyl­pyrrolidin-2-one-κ*O*)calcium(II)]-μ-(*E*)-1,1′-dimethyl-2,2′-dioxo-1,1′,2,2′-tetra­hydro-[3,3′-bipyrrolyl­idene]-5,5′-bis­(thiol­ato)-κ^2^ *O*:*O*′], [Ca(C_10_H_8_N_2_O_2_S_2_)(C_5_H_9_NO)_4_]_*n*_, **1**, crystallizes in the triclinic space group *P* . The crystal studied was twinned by non-merohedry *via* two different twofold operations, about the normals to (001) and (10), giving four twin domains with refined occupancies of 0.412 (4), 0.366 (4), 0.055 (1), 0.167 (4). The Ca atoms are located on centers of inversion. Each Ca is surrounded by four 1-methyl­pyrrolidin-2-one (NMP) ligands and coordinated through one of the two O atoms to two (*E*)-1,1′-dimethyl-2,2′-dioxo-1,1′,2,2′-tetra­hydro-[3,3′-bipyrrolyl­idene]-5,5′-bis­(thiol­ate), [C_10_H_8_N_2_O_2_S_2_]^2−^, dianions (abbreviation: DMTBT). This dianion thus facilitates the formation of a 1-D polymer, which propagates in the [011] direction. These ribbons are linked by inter­molecular C—H⋯S inter­actions. Each Ca atom is in an octa­hedral CaO_6_ six-coordinate environment with Ca—O bond lengths ranging from 2.308 (6) to 2.341 (6) Å, *cis* bond angles ranging from 88.2 (2) to 91.8 (2)° and the *trans* angles all 180° due to the Ca atoms being located on centers of inversion. Theoretical calculations were carried out using density functional theory (DFT) and the results showed that although the central DMTBT dianion is planar there is likely some resonance across the central bond between both aza­pentyl rings, but this is not sufficient to establish a ring current. The calculated UV–vis spectrum shows a peak at 625 nm, which accounts for the deep blue–purple color of solutions of the complex.

## Abstract 2

The synthesis, structure, and theoretical studies of a calcium complex of a unique dianion derived from *N*-methyl-2-pyrrolidine are explored.

## Chemical context   

There has been recent inter­est in ternary sulfides as two-color IR optical window materials (Jarý *et al.*, 2015 ▸) as well as other uses, such as phosphor materials (Sun *et al.*, 1994 ▸). In the synthesis of alkaline earth ternary sulfides, reactions using metal thiol­ates and H_2_S are an obvious avenue of study. An obstacle to such work is the lack of soluble alkaline earth thiol­ates. As part of a program for the investigation of precursors for the synthesis of a wide variety of metal sulfide materials, the reactions between alkaline earth amides and thiol­ate ligands were explored (Purdy *et al.*, 1997 ▸). When the barium complex, Ba(SCMe_3_)_2_, was crystallized from a mixed NMP solution over a period of years, an unusual barium sulfur cluster was obtained, [Ba_6_(C_4_H_9_S)_10_S(C_5_H_9_NO)_6_], containing a central μ_6_-sulfido atom surrounded by six Ba atoms and NMP ligands (Butcher & Purdy, 2006 ▸). On the other hand, when solutions of the analogous calcium complex are substituted, these solutions turn blue over time (or more quickly when heated).
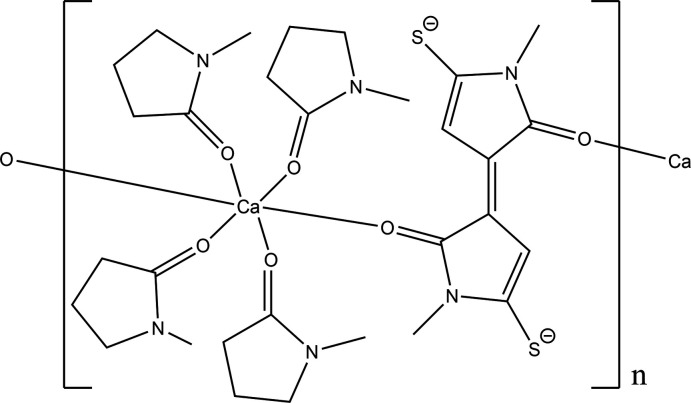


The solvent NMP, along with the presence of calcium ions, appears to play a crucial role in this reactivity. Solutions of calcium ions in *N*-methyl-2-pyrrolidine have shown unusual reactivity in many areas, including the synthesis of thermally stable polyamides (Mallakpour & Kolahdoozan, 2008 ▸; Faghihi, 2009 ▸; Faghihi *et al.*, 2010 ▸; Dewilde *et al.*, 2016 ▸), the synthesis and structural studies of functional coordination polymers from calcium carboxyl­ates based on cluster- and rod-like building blocks (Kang *et al.*, 2014 ▸), dental applications using calcium hydroxide paste along with NMP (Lim *et al.*, 2017 ▸; Kim *et al.*, 2020 ▸), the formulation of solid self-nanoemulsifying drug-delivery systems (Agrawal *et al.*, 2015 ▸), and in lyotropic liquid crystalline behavior of poly(2-cyano-*p*-phenyl­ene terephthalamide) in *N*-methyl-2-pyrrolidone/calcium chloride solutions (Jung *et al.*, 2016 ▸).

The results of this unusual reactivity are explored in this paper.

## Structural commentary   

The title compound, C_30_H_44_CaN_6_O_6_S_2_, **1**, crystallizes with the triclinic space group, *P* 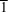. The Ca atoms are located on centers of inversion. Each Ca atom is surrounded by four NMP ligands and coordinated through one of the two O atoms to two DMTBT dianions. This dianion thus results in the formation of a 1-D polymer, which extends in the [011] direction. Each Ca atom is in a CaO_6_ six-coordinate environment with Ca—O bond lengths ranging from 2.308 (6) to 2.341 (6) Å, *cis* bond angles ranging from 88.2 (2) to 91.8 (2)° and the *trans* angles all 180° due to the Ca atoms being located on centers of inversion. Thus each Ca atom has close to ideal octa­hedral geometry.

**Figure 1:**
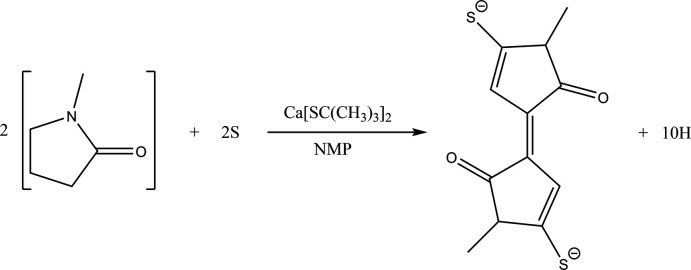
Diagram showing how the dianion has resulted from the condensation of two mol­ecules of NMP along with the incorporation of two sulfur atoms in the form of C—S^−^ bonds.

In view of the inter­est in combinations of NMP with Ca ions as a reaction medium, it is surprising to note that in the literature (Kang *et al.*, 2014 ▸; Qinghua, 2018 ▸) there are only three instances of structures containing Ca coordinated to NMP. In these structures, the Ca—O bond length varies from 2.244 (4) to 2.305 (3) Å, which match the values in **1**. However, there are no previous structures containing the dianion or any related species. This dianion has resulted from the condensation of two mol­ecules of NMP along with the incorporation of two sulfur atoms in the form of C—S^−^ bonds (Fig. 1 ▸). In view of the reactivity of Ca in NMP solutions as mentioned above, it appears that the calcium associated with NMP templates this reaction.

**Figure 2:**
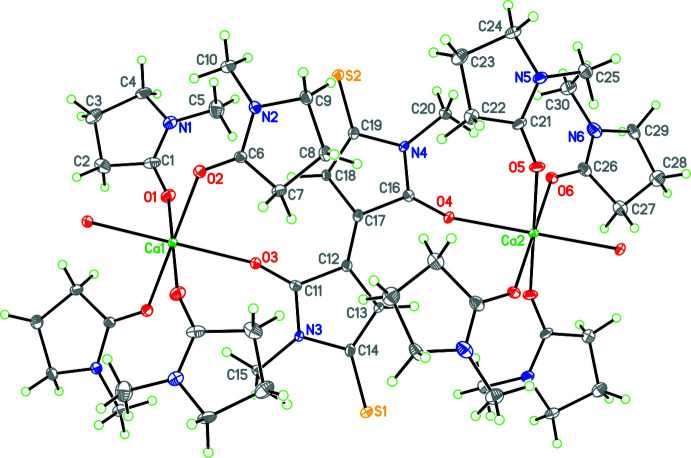
Diagram showing the dianion linking the Ca centers and showing atom labeling for the asymmetric unit. Atomic displacement parameters are at the 30% probability level.

The two five-membered rings of the dianion (Fig. 2 ▸) are planar (r.m.s. deviations for C11 to N3 and C16 to N4 of 0.005 and 0.009 Å, respectively) and the two rings are almost coplanar [dihedral angle between rings of only 1.0 (5)°]. The two nitro­gen atoms in the ring are almost trigonal [sum of angles about N3 and N4 of 359.5 (7) and 359.8 (7)°, respectively] with their attached methyl groups being only 0.157 (15) and 0.051 (15) Å out of the plane of their respective rings. Thus there must be considerable aromatic character in the linked five-membered rings of the dianion. The bond order of both the C—O and C—S moieties in both rings appear to be close to double bond in character with distances of 1.242 (9) and 1.256 (10) Å for C—O and 1.696 (9) and 1.713 (9) Å for C—S (Trinajstić, 1968 ▸).

## DFT calculations   

**Figure 3:**
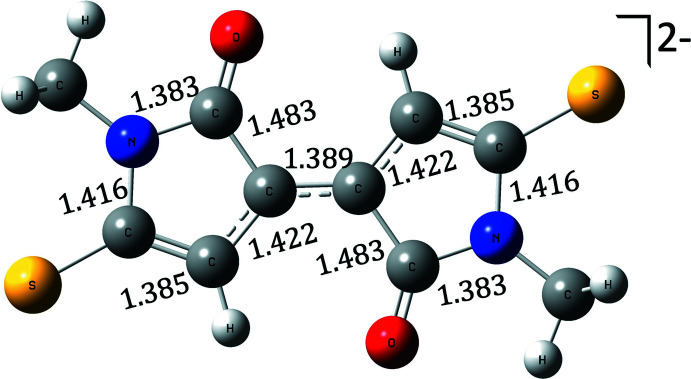
Ground state equilibrium structure for the DMTBTdianion. The bond lengths, in units of Å, are overlaid.

**Figure 4:**
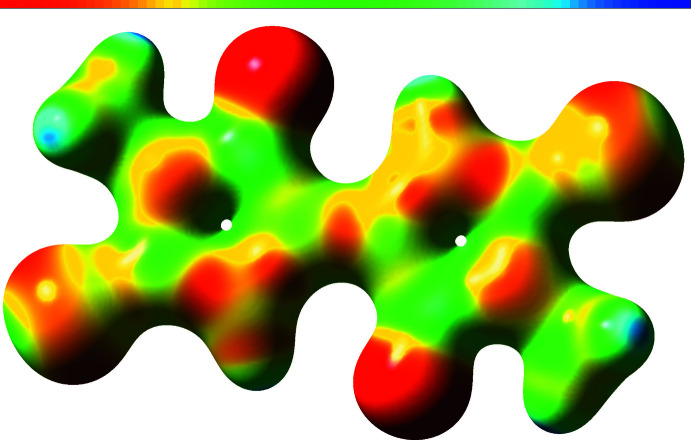
Ground state charge density for the DMTBT dianion. The electric potential ranges from −0.2 atomic units (red) to 0.2 atomic units (blue).

**Figure 5:**
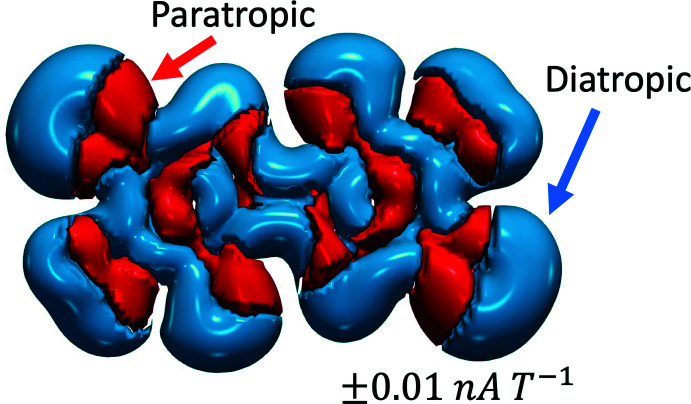
Signed modulus of the magnetically induced current density in the DMTBT dianion. Note, the total diatropic current is found to be 9.53 *na T* ^−1^, paratropic is −8.44 *na T* ^−1^, and total is 1.08 *na T* ^−1^.

**Figure 6:**
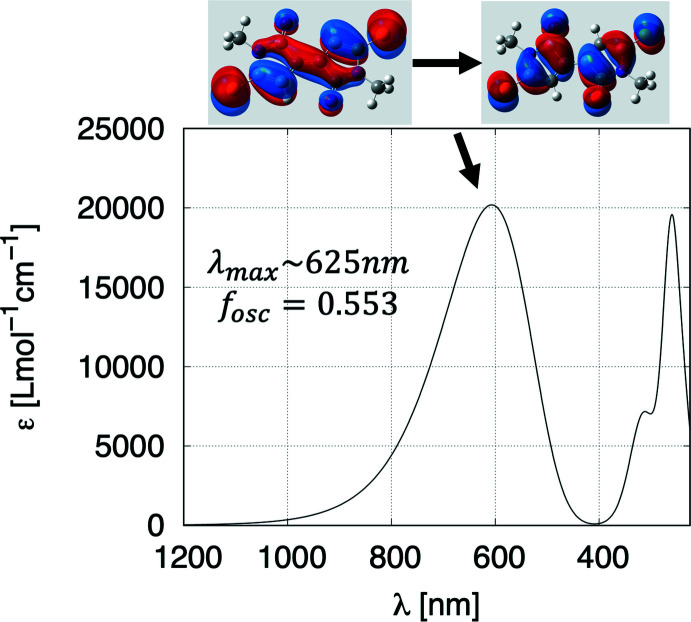
Calculated UV–vis spectrum of the DMTBT dianion from TD-SCF. The HOMO–LUMO states featuring the dominant transition are shown above the spectrum.

The calculations for the DMTBT dianions were treated with density functional theory (DFT) within the *Gaussian 09* suite (Frisch *et al.*, 2016 ▸; Hohenberg & Kohn, 1964 ▸). To approximate the exchange-correlation functional for this compound we used the Heyd–Scuseria–Ernzerhof (HSE) screened hybrid HSE06 functional within an unrestricted self-consistent field for the singlet dianion ground state (Heyd *et al.*, 2005 ▸). The elements composing the compound are expanded in the 6-311+G(d,p) Gaussian basis set, which is included in the geometry optimization with tight convergence criteria and ultrafine integration grid (McLean & Chandler, 1980 ▸; Curtiss *et al.*, 1995 ▸). The ground-state equilibrium structure for the dianion state is shown in Fig. 3 ▸ with bond lengths in Å overlaid. The optimized geometry was used in all subsequent calculations. The charge distribution is shown in Fig. 4 ▸ and from this it can be seen that the negative charge is distributed between the S and O atoms, with the O atom having the major part in each ring. To understand aromaticity in this compound, the ring currents were computed starting from the gauge-independent atomic orbitals (GIAO) method (London, 1937 ▸; Cheeseman *et al.*, 1996 ▸). The GIAO results were used to generate the signed modulus of the current density and average induced current with gauge-including magnetically induced current code (GIMIC) on a dense grid (Johansson *et al.*, 2005 ▸; Taubert *et al.*, 2008 ▸; Fliegl *et al.*, 2009 ▸, 2011 ▸, 2015 ▸, 2016 ▸). The results are shown in Fig. 5 ▸ and show that there likely is some resonance across the central bond between both aza­pentyl rings, but this is not sufficient to establish a ring current (Peeks *et al.*, 2017 ▸). The UV–vis spectrum (Fig. 6 ▸) is computed with time-dependent self-consistent density functional theory (TD-SCF) with 1000 additional states (Casida *et al.*, 1998 ▸; Furche & Ahlrichs, 2002 ▸). This shows a peak at 625 nm, originating from the HOMO–LUMO transition (Fig. 6 ▸), which accounts for the deep blue–purple color of solutions of the complex. The experimental λ_max_ of the blue solution is at 671 nm, which may include colored compounds besides the title compound, as what crystallizes is not necessarily representative of the remaining solution. Thus, while we could obtain a spectrum similar to that generated from calculations, we cannot be sure that what is in solution is the same material that is in the crystals. The oxidation of NMP to the title dianion requires removal of ten hydrogen atoms, and this process must involve multiple steps that produce many different inter­mediates. An attempt to prepare this dianion by oxidation of NMP with S_8_ in the presence of CaS under an inert atmosphere produced purple- and blue-colored compounds, which have yet to be identified.

## Supra­molecular features   

**Figure 7:**
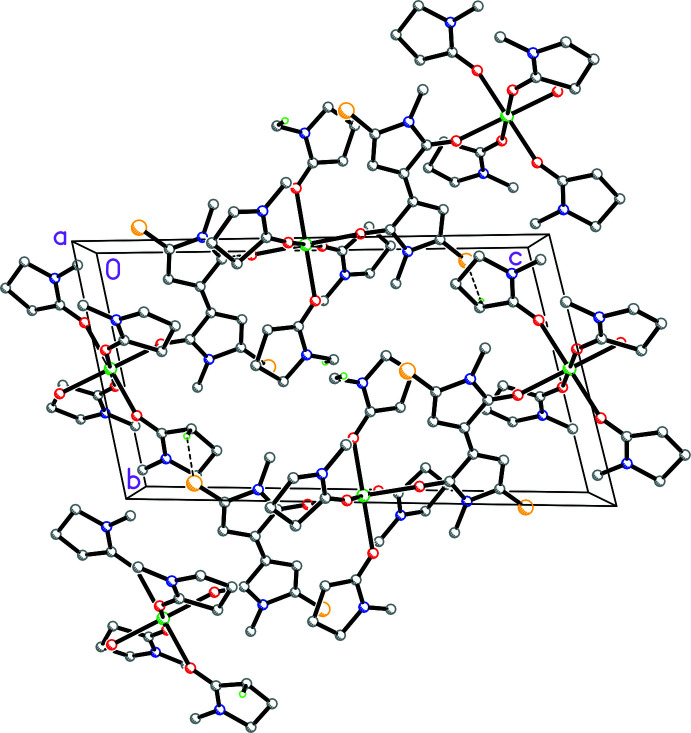
Diagram showing how the dianion links the Ca centers into ribbons in the [011] direction. All hydrogen atoms omitted except those involved in C—H⋯S inter­actions. Dashed lines indicate the inter-ribbon C—H⋯S inter­actions linking these ribbons.

The Ca atoms are located on centers of inversion. Each Ca is surrounded by 4 NMP ligands and coordinated through one of the two O atoms to two DMTBT dianions. This dianion thus facilitates the formation of 1-D ribbons, which propagate in the [011] direction. These ribbons are linked by C—H⋯S inter­actions (Table 1 ▸), as shown in Fig. 7 ▸.

## Database survey   

A search of the Cambridge Structural Database [CSD version 5.41 (November 2019); Groom *et al.*, 2016 ▸] for both dianion and structures containing NMP coordinated to Ca gave only three examples of the latter [POMSER and POMSOB (Kang *et al.*, 2014 ▸); WIMBIG (Qinghua, 2018 ▸)] and no examples of the former.

## Synthesis and crystallization   

Ca(SCMe_3_)_2_ (Purdy *et al.*, 1997 ▸) was dissolved in *N*-methyl-2-pyrrolidone containing about 10% C_6_D_6_ and a drop of tetra­methyl­silane and sealed in an NMR tube. After ∼6.5 years, a mass of deep-blue crystals was discovered in the NMR tube. One was selected and transferred to the cold stream of the diffractometer at 100 K. While perfectly stable under an inert atmosphere, the color changes in a few minutes after exposure to air. ^13^C NMR spectra of the solution showed nothing that can be attributed to the title compound, so it is likely that the concentration is too low to be observed. A UV–vis spectrum of the solution showed a λ_max_ at 671 nm. For an attempt to use Ca to template the sulfur oxidation of NMP, 0.53 g of CaS, 0.54 g of S_8_, and 20 mL of dry NMP were stirred in an H-tube for 373 K under N_2_ for 3 d. A pink–purple solution formed, but turned blue when the filtered solution was heated over CaS, allowing H_2_S to escape, and then turned pink again when concentrated.

## Refinement details   

Crystal data, data collection and structure refinement details are summarized in Table 2 ▸. All hydrogen atoms for the major component were located in difference Fourier maps and included in idealized positions using a riding model with atomic displacement parameters of *U*_iso_(H) = 1.2*U*_eq_(C, N) [1.5*U*_eq_(C) for CH_3_], with C—H distances ranging from 0.95 to 0.99 Å. The crystal was twinned by non-merohedry *via* two different twofold operations, about the normals to (001) and (1
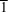
0), giving four twin domains with refined occupancies of 0.412 (4), 0.366 (4), 0.055 (1), 0.167 (4).

## Supplementary Material

Crystal structure: contains datablock(s) I. DOI: 10.1107/S205698902001628X/pk2649sup1.cif

Structure factors: contains datablock(s) I. DOI: 10.1107/S205698902001628X/pk2649Isup3.hkl

CCDC reference: 2050442

Additional supporting information:  crystallographic information; 3D view; checkCIF report

## Figures and Tables

**Table 1:** Hydrogen-bond geometry (Å, °)

*D*—H⋯*A*	*D*—H	H⋯*A*	*D*⋯*A*	*D*—H⋯*A*
C4—H4*A*⋯O3^i^	0.99	2.51	3.456 (12)	159
C10—H10*B*⋯S2	0.98	2.97	3.820 (10)	146
C15—H15*A*⋯O2^ii^	0.98	2.58	3.545 (10)	167
C20—H20*A*⋯O6	0.98	2.53	3.504 (11)	172
C22—H22*A*⋯S1^iii^	0.99	2.93	3.839 (11)	154
C23—H23*B*⋯N3^iii^	0.99	2.69	3.655 (12)	165
C27—H27*A*⋯O4^iv^	0.99	2.63	3.444 (11)	140

Symmetry codes: (i) 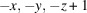; (ii) 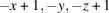; (iii) 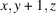; (iv) 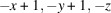.

**Table 2:** Experimental details

Crystal data	
Chemical formula	[Ca(C_10_H_8_N_2_O_2_S_2_)(C_5_H_9_NO)_4_]
*M*_r_	688.91
Crystal system, space group	Triclinic, *P* 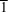
Temperature (K)	100
*a*, *b*, *c* (Å)	8.6686 (13), 10.5190 (15), 18.998 (3)
α, β, γ (°)	75.488 (9), 76.847 (7), 80.905 (6)
*V* (Å^3^)	1623.6 (4)
*Z*	2
Radiation type	Mo *K*α
μ (mm^−1^)	0.37
Crystal size (mm)	0.25 × 0.21 × 0.09

Data collection	
Diffractometer	Bruker APEXII CCD
Absorption correction	Multi-scan (*SADABS*; Sheldrick, 1996 ▸)
*T*_min_, *T*_max_	0.569, 0.745
No. of measured, independent and observed [*I* > 2σ(*I*)] reflections	8230, 8230, 5186
*R*_int_	0.083
(sin θ/λ)_max_ (Å^−1^)	0.667

Refinement	
*R*[*F* ^2^ > 2σ(*F* ^2^)], *wR*(*F* ^2^), *S*	0.111, 0.301, 1.06
No. of reflections	8230
No. of parameters	418
H-atom treatment	H-atom parameters constrained
Δρ_max_, Δρ_min_ (e Å^−3^)	1.14, −0.70

Computer programs: *APEX2* (Bruker, 2005 ▸), *SAINT* (Bruker, 2002 ▸), *SHELXT* (Sheldrick 2015*a* ▸), *SHELXL2018/3* (Sheldrick, 2015*b* ▸) and *SHELXTL* (Sheldrick 2008 ▸).

## supplementary crystallographic information

### Crystal data

**Table:**

[Ca(C_10_H_8_N_2_O_2_S_2_)(C_5_H_9_NO)_4_]	*Z* = 2
*M**_r_* = 688.91	*F*(000) = 732
Triclinic, *P*1	*D*_x_ = 1.409 Mg m^−^^3^
*a* = 8.6686 (13) Å	Mo *K*α radiation, λ = 0.71073 Å
*b* = 10.5190 (15) Å	Cell parameters from 4441 reflections
*c* = 18.998 (3) Å	θ = 2.8–28.1°
α = 75.488 (9)°	µ = 0.37 mm^−^^1^
β = 76.847 (7)°	*T* = 100 K
γ = 80.905 (6)°	Plate, blue-crimson
*V* = 1623.6 (4) Å^3^	0.25 × 0.21 × 0.09 mm

### Data collection

**Table:**

Bruker APEXII CCD diffractometer	5186 reflections with *I* > 2σ(*I*)
φ and ω scans	*R*_int_ = 0.083
Absorption correction: multi-scan (SADABS; Sheldrick, 1996)	θ_max_ = 28.3°, θ_min_ = 2.5°
*T*_min_ = 0.569, *T*_max_ = 0.745	*h* = −11→11
8230 measured reflections	*k* = −13→14
8230 independent reflections	*l* = 0→25

### Refinement

**Table:**

Refinement on *F*^2^	Primary atom site location: structure-invariant direct methods
Least-squares matrix: full	Secondary atom site location: difference Fourier map
*R*[*F*^2^ > 2σ(*F*^2^)] = 0.111	Hydrogen site location: inferred from neighbouring sites
*wR*(*F*^2^) = 0.301	H-atom parameters constrained
*S* = 1.06	*w* = 1/[σ^2^(*F*_o_^2^) + 10.0576*P*] where *P* = (*F*_o_^2^ + 2*F*_c_^2^)/3
8230 reflections	(Δ/σ)_max_ < 0.001
418 parameters	Δρ_max_ = 1.14 e Å^−^^3^
0 restraints	Δρ_min_ = −0.70 e Å^−^^3^

### Special details

**Table:**

Geometry. All esds (except the esd in the dihedral angle between two l.s. planes) are estimated using the full covariance matrix. The cell esds are taken into account individually in the estimation of esds in distances, angles and torsion angles; correlations between esds in cell parameters are only used when they are defined by crystal symmetry. An approximate (isotropic) treatment of cell esds is used for estimating esds involving l.s. planes.
Refinement. Refined as a four-component twin

### Fractional atomic coordinates and isotropic or equivalent isotropic displacement parameters (Å^2^)

**Table:**

	*x*	*y*	*z*	*U*_iso_*/*U*_eq_	
Ca1	0.500000	0.000000	0.500000	0.0209 (5)	
Ca2	0.500000	0.500000	0.000000	0.0207 (5)	
S1	0.8711 (3)	−0.0571 (2)	0.14841 (12)	0.0295 (5)	
S2	0.0342 (3)	0.4917 (2)	0.34997 (12)	0.0309 (5)	
O1	0.2279 (7)	−0.0055 (7)	0.5364 (3)	0.0338 (15)	
O2	0.4684 (7)	0.2267 (6)	0.4928 (3)	0.0296 (13)	
O3	0.4856 (7)	0.0391 (6)	0.3747 (3)	0.0262 (13)	
O4	0.4201 (7)	0.3985 (6)	0.1239 (3)	0.0247 (13)	
O5	0.5191 (7)	0.6900 (6)	0.0382 (3)	0.0310 (14)	
O6	0.2373 (7)	0.5739 (6)	−0.0113 (3)	0.0283 (13)	
N1	−0.0137 (10)	0.1047 (8)	0.5747 (4)	0.0386 (19)	
N2	0.4026 (9)	0.4436 (7)	0.4464 (4)	0.0258 (15)	
N3	0.6702 (7)	−0.0260 (6)	0.2776 (3)	0.0182 (13)	
N4	0.2341 (8)	0.4610 (7)	0.2207 (3)	0.0216 (14)	
N5	0.3706 (9)	0.8684 (7)	0.0771 (4)	0.0291 (16)	
N6	0.0237 (10)	0.6894 (8)	−0.0595 (5)	0.042 (2)	
C1	0.0984 (11)	0.0092 (10)	0.5796 (6)	0.036 (2)	
C2	0.0476 (12)	−0.0938 (10)	0.6544 (6)	0.041 (2)	
H2A	0.016867	−0.173904	0.645037	0.049*	
H2B	0.134884	−0.119763	0.682601	0.049*	
C3	−0.0935 (13)	−0.0196 (11)	0.6954 (6)	0.044 (3)	
H3A	−0.061227	0.016484	0.732660	0.053*	
H3B	−0.178066	−0.077899	0.720847	0.053*	
C4	−0.1524 (11)	0.0942 (11)	0.6338 (5)	0.039 (2)	
H4A	−0.243478	0.071000	0.617940	0.047*	
H4B	−0.184156	0.177675	0.651072	0.047*	
C5	−0.0042 (15)	0.2138 (12)	0.5117 (6)	0.054 (3)	
H5A	0.087633	0.194829	0.473425	0.081*	
H5B	0.008172	0.293686	0.526633	0.081*	
H5C	−0.101935	0.227553	0.491797	0.081*	
C6	0.4926 (10)	0.3304 (8)	0.4429 (5)	0.0241 (16)	
C7	0.6206 (10)	0.3457 (9)	0.3749 (5)	0.0289 (18)	
H7A	0.617176	0.280747	0.345683	0.035*	
H7B	0.726897	0.332815	0.388168	0.035*	
C8	0.5860 (13)	0.4870 (9)	0.3308 (5)	0.039 (2)	
H8A	0.556192	0.486307	0.283647	0.047*	
H8B	0.680617	0.535770	0.319590	0.047*	
C9	0.4453 (10)	0.5516 (9)	0.3816 (5)	0.0298 (19)	
H9A	0.478478	0.625278	0.396386	0.036*	
H9B	0.354412	0.585444	0.356079	0.036*	
C10	0.2645 (10)	0.4624 (9)	0.5035 (5)	0.033 (2)	
H10A	0.262799	0.385681	0.545334	0.049*	
H10B	0.167597	0.472303	0.483435	0.049*	
H10C	0.269587	0.542061	0.520446	0.049*	
C11	0.5490 (9)	0.0573 (8)	0.3071 (5)	0.0207 (16)	
C12	0.5132 (10)	0.1665 (8)	0.2445 (4)	0.0227 (16)	
C13	0.6225 (10)	0.1396 (8)	0.1799 (4)	0.0229 (17)	
H13A	0.629740	0.193468	0.131270	0.027*	
C14	0.7155 (10)	0.0228 (8)	0.1998 (4)	0.0236 (17)	
C15	0.7302 (10)	−0.1537 (8)	0.3191 (5)	0.0244 (17)	
H15A	0.685079	−0.162070	0.372077	0.037*	
H15B	0.846621	−0.160100	0.311137	0.037*	
H15C	0.699104	−0.224643	0.301992	0.037*	
C16	0.3566 (9)	0.3793 (8)	0.1907 (4)	0.0207 (16)	
C17	0.3937 (9)	0.2709 (8)	0.2528 (4)	0.0217 (16)	
C18	0.2858 (9)	0.2971 (8)	0.3173 (4)	0.0189 (15)	
H18A	0.282092	0.243790	0.365955	0.023*	
C19	0.1869 (10)	0.4118 (8)	0.2988 (5)	0.0241 (17)	
C20	0.1567 (10)	0.5776 (8)	0.1775 (5)	0.0268 (18)	
H20A	0.172438	0.568900	0.126140	0.040*	
H20B	0.203080	0.656072	0.178620	0.040*	
H20C	0.042424	0.586495	0.198735	0.040*	
C21	0.4733 (10)	0.7619 (9)	0.0845 (5)	0.0280 (18)	
C22	0.5330 (12)	0.7414 (10)	0.1554 (5)	0.038 (2)	
H22A	0.641539	0.768551	0.145754	0.045*	
H22B	0.533749	0.648017	0.182951	0.045*	
C23	0.4100 (12)	0.8321 (10)	0.1980 (5)	0.038 (2)	
H23A	0.324693	0.781927	0.232182	0.045*	
H23B	0.461434	0.873266	0.226868	0.045*	
C24	0.3430 (11)	0.9365 (10)	0.1375 (5)	0.034 (2)	
H24A	0.227943	0.962313	0.153860	0.041*	
H24B	0.400454	1.016203	0.122862	0.041*	
C25	0.2933 (13)	0.9182 (10)	0.0144 (6)	0.042 (2)	
H25A	0.313195	0.852532	−0.016107	0.064*	
H25B	0.335889	1.000203	−0.015339	0.064*	
H25C	0.178354	0.935811	0.032024	0.064*	
C26	0.1555 (11)	0.6123 (9)	−0.0607 (5)	0.033 (2)	
C27	0.1989 (12)	0.5684 (9)	−0.1368 (5)	0.035 (2)	
H27A	0.312863	0.574708	−0.159708	0.042*	
H27B	0.174354	0.477044	−0.130614	0.042*	
C28	0.0932 (13)	0.6667 (12)	−0.1818 (6)	0.045 (3)	
H28A	0.051692	0.623984	−0.213478	0.055*	
H28B	0.152731	0.740055	−0.213860	0.055*	
C29	−0.0437 (12)	0.7182 (10)	−0.1257 (6)	0.040 (2)	
H29A	−0.073001	0.814245	−0.142362	0.048*	
H29B	−0.138893	0.671270	−0.117271	0.048*	
C30	−0.0501 (13)	0.7456 (11)	0.0025 (6)	0.044 (2)	
H30A	−0.007574	0.695702	0.046247	0.066*	
H30B	−0.165465	0.741933	0.012262	0.066*	
H30C	−0.027947	0.837780	−0.008253	0.066*	

### Atomic displacement parameters (Å^2^)

**Table:**

	*U*^11^	*U*^22^	*U*^33^	*U*^12^	*U*^13^	*U*^23^
Ca1	0.0226 (11)	0.0206 (11)	0.0142 (10)	0.0017 (9)	0.0007 (8)	−0.0005 (8)
Ca2	0.0218 (11)	0.0214 (11)	0.0161 (10)	0.0035 (9)	−0.0017 (8)	−0.0044 (8)
S1	0.0307 (11)	0.0307 (11)	0.0220 (10)	0.0046 (9)	0.0012 (8)	−0.0072 (8)
S2	0.0275 (11)	0.0376 (12)	0.0226 (10)	0.0054 (9)	−0.0006 (8)	−0.0069 (9)
O1	0.030 (3)	0.037 (4)	0.032 (3)	−0.005 (3)	0.003 (3)	−0.011 (3)
O2	0.039 (3)	0.023 (3)	0.024 (3)	0.000 (3)	−0.003 (3)	−0.004 (2)
O3	0.029 (3)	0.025 (3)	0.020 (3)	0.003 (2)	−0.001 (2)	−0.002 (2)
O4	0.028 (3)	0.028 (3)	0.015 (3)	0.000 (2)	0.000 (2)	−0.004 (2)
O5	0.033 (3)	0.028 (3)	0.033 (3)	0.006 (3)	−0.003 (3)	−0.017 (3)
O6	0.024 (3)	0.029 (3)	0.027 (3)	0.008 (2)	−0.007 (2)	−0.004 (2)
N1	0.039 (5)	0.042 (5)	0.032 (4)	0.004 (4)	−0.002 (3)	−0.012 (4)
N2	0.029 (4)	0.022 (3)	0.024 (3)	−0.001 (3)	−0.003 (3)	−0.002 (3)
N3	0.021 (3)	0.020 (3)	0.012 (3)	−0.001 (3)	−0.002 (2)	−0.003 (2)
N4	0.024 (3)	0.023 (3)	0.015 (3)	−0.001 (3)	0.003 (3)	−0.005 (3)
N5	0.032 (4)	0.031 (4)	0.026 (4)	0.002 (3)	−0.004 (3)	−0.014 (3)
N6	0.037 (5)	0.036 (5)	0.049 (5)	0.000 (4)	−0.015 (4)	−0.003 (4)
C1	0.024 (4)	0.040 (5)	0.050 (6)	0.000 (4)	−0.004 (4)	−0.026 (5)
C2	0.035 (5)	0.037 (5)	0.058 (6)	−0.004 (4)	−0.022 (5)	−0.013 (5)
C3	0.040 (6)	0.055 (7)	0.043 (6)	−0.016 (5)	0.001 (5)	−0.024 (5)
C4	0.025 (4)	0.052 (6)	0.044 (6)	0.008 (4)	−0.004 (4)	−0.028 (5)
C5	0.061 (8)	0.050 (7)	0.047 (6)	−0.016 (6)	−0.007 (6)	0.001 (5)
C6	0.028 (4)	0.022 (4)	0.026 (4)	0.000 (3)	−0.009 (3)	−0.010 (3)
C7	0.027 (4)	0.031 (5)	0.030 (4)	0.002 (4)	−0.004 (3)	−0.013 (4)
C8	0.053 (6)	0.029 (5)	0.028 (5)	−0.002 (4)	−0.001 (4)	0.002 (4)
C9	0.029 (4)	0.030 (5)	0.028 (4)	0.000 (4)	−0.008 (4)	−0.002 (4)
C10	0.029 (5)	0.035 (5)	0.034 (5)	−0.001 (4)	−0.002 (4)	−0.014 (4)
C11	0.021 (4)	0.018 (4)	0.027 (4)	−0.002 (3)	−0.008 (3)	−0.008 (3)
C12	0.027 (4)	0.023 (4)	0.019 (4)	−0.002 (3)	−0.006 (3)	−0.005 (3)
C13	0.029 (4)	0.023 (4)	0.015 (3)	0.004 (3)	−0.002 (3)	−0.006 (3)
C14	0.032 (4)	0.021 (4)	0.017 (4)	−0.007 (3)	−0.003 (3)	−0.002 (3)
C15	0.027 (4)	0.018 (4)	0.028 (4)	0.001 (3)	−0.009 (3)	−0.005 (3)
C16	0.016 (4)	0.022 (4)	0.024 (4)	−0.006 (3)	−0.003 (3)	−0.002 (3)
C17	0.021 (4)	0.028 (4)	0.017 (4)	−0.004 (3)	−0.007 (3)	−0.004 (3)
C18	0.018 (3)	0.020 (4)	0.015 (3)	−0.001 (3)	−0.002 (3)	0.000 (3)
C19	0.024 (4)	0.026 (4)	0.022 (4)	−0.004 (3)	−0.004 (3)	−0.003 (3)
C20	0.026 (4)	0.025 (4)	0.028 (4)	0.002 (3)	−0.006 (3)	−0.005 (3)
C21	0.029 (4)	0.031 (5)	0.024 (4)	−0.003 (4)	0.004 (3)	−0.015 (3)
C22	0.039 (5)	0.038 (5)	0.033 (5)	−0.001 (4)	−0.005 (4)	−0.007 (4)
C23	0.035 (5)	0.050 (6)	0.034 (5)	−0.008 (4)	−0.006 (4)	−0.018 (4)
C24	0.034 (5)	0.036 (5)	0.032 (5)	0.001 (4)	0.000 (4)	−0.018 (4)
C25	0.043 (6)	0.042 (6)	0.035 (5)	0.011 (5)	−0.011 (4)	−0.002 (4)
C26	0.031 (5)	0.023 (4)	0.041 (5)	−0.006 (4)	−0.007 (4)	0.000 (4)
C27	0.036 (5)	0.031 (5)	0.043 (5)	−0.010 (4)	−0.014 (4)	−0.007 (4)
C28	0.042 (6)	0.055 (7)	0.043 (6)	−0.005 (5)	−0.013 (5)	−0.013 (5)
C29	0.039 (5)	0.036 (5)	0.042 (5)	0.004 (4)	−0.022 (4)	0.003 (4)
C30	0.042 (6)	0.049 (6)	0.044 (6)	−0.003 (5)	−0.007 (5)	−0.020 (5)

### Geometric parameters (Å, º)

**Table:**

Ca1—O1	2.308 (6)	C7—C8	1.533 (12)
Ca1—O1^i^	2.308 (6)	C7—H7A	0.9900
Ca1—O2	2.329 (6)	C7—H7B	0.9900
Ca1—O2^i^	2.329 (6)	C8—C9	1.552 (13)
Ca1—O3^i^	2.341 (6)	C8—H8A	0.9900
Ca1—O3	2.341 (6)	C8—H8B	0.9900
Ca2—O4^ii^	2.322 (5)	C9—H9A	0.9900
Ca2—O4	2.322 (5)	C9—H9B	0.9900
Ca2—O6	2.327 (6)	C10—H10A	0.9800
Ca2—O6^ii^	2.327 (6)	C10—H10B	0.9800
Ca2—O5^ii^	2.331 (6)	C10—H10C	0.9800
Ca2—O5	2.331 (6)	C11—C12	1.483 (11)
S1—C14	1.713 (9)	C12—C17	1.399 (10)
S2—C19	1.696 (9)	C12—C13	1.429 (11)
O1—C1	1.244 (11)	C13—C14	1.376 (11)
O2—C6	1.264 (10)	C13—H13A	0.9500
O3—C11	1.256 (10)	C15—H15A	0.9800
O4—C16	1.242 (9)	C15—H15B	0.9800
O5—C21	1.258 (10)	C15—H15C	0.9800
O6—C26	1.253 (11)	C16—C17	1.475 (11)
N1—C1	1.283 (12)	C17—C18	1.422 (11)
N1—C5	1.433 (13)	C18—C19	1.382 (11)
N1—C4	1.444 (12)	C18—H18A	0.9500
N2—C6	1.324 (10)	C20—H20A	0.9800
N2—C10	1.446 (11)	C20—H20B	0.9800
N2—C9	1.473 (11)	C20—H20C	0.9800
N3—C11	1.369 (10)	C21—C22	1.506 (13)
N3—C14	1.422 (9)	C22—C23	1.540 (13)
N3—C15	1.460 (10)	C22—H22A	0.9900
N4—C16	1.369 (10)	C22—H22B	0.9900
N4—C19	1.430 (10)	C23—C24	1.528 (14)
N4—C20	1.453 (10)	C23—H23A	0.9900
N5—C21	1.315 (11)	C23—H23B	0.9900
N5—C25	1.443 (12)	C24—H24A	0.9900
N5—C24	1.456 (11)	C24—H24B	0.9900
N6—C26	1.291 (12)	C25—H25A	0.9800
N6—C30	1.426 (13)	C25—H25B	0.9800
N6—C29	1.450 (12)	C25—H25C	0.9800
C1—C2	1.576 (15)	C26—C27	1.573 (14)
C2—C3	1.510 (14)	C27—C28	1.501 (14)
C2—H2A	0.9900	C27—H27A	0.9900
C2—H2B	0.9900	C27—H27B	0.9900
C3—C4	1.562 (15)	C28—C29	1.532 (15)
C3—H3A	0.9900	C28—H28A	0.9900
C3—H3B	0.9900	C28—H28B	0.9900
C4—H4A	0.9900	C29—H29A	0.9900
C4—H4B	0.9900	C29—H29B	0.9900
C5—H5A	0.9800	C30—H30A	0.9800
C5—H5B	0.9800	C30—H30B	0.9800
C5—H5C	0.9800	C30—H30C	0.9800
C6—C7	1.492 (12)		
			
O1—Ca1—O1^i^	180.0	N2—C9—H9B	110.9
O1—Ca1—O2	90.3 (2)	C8—C9—H9B	110.9
O1^i^—Ca1—O2	89.7 (2)	H9A—C9—H9B	108.9
O1—Ca1—O2^i^	89.7 (2)	N2—C10—H10A	109.5
O1^i^—Ca1—O2^i^	90.3 (2)	N2—C10—H10B	109.5
O2—Ca1—O2^i^	180.0 (3)	H10A—C10—H10B	109.5
O1—Ca1—O3^i^	88.3 (2)	N2—C10—H10C	109.5
O1^i^—Ca1—O3^i^	91.7 (2)	H10A—C10—H10C	109.5
O2—Ca1—O3^i^	89.9 (2)	H10B—C10—H10C	109.5
O2^i^—Ca1—O3^i^	90.1 (2)	O3—C11—N3	124.0 (7)
O1—Ca1—O3	91.7 (2)	O3—C11—C12	129.5 (7)
O1^i^—Ca1—O3	88.3 (2)	N3—C11—C12	106.5 (7)
O2—Ca1—O3	90.1 (2)	C17—C12—C13	130.6 (7)
O2^i^—Ca1—O3	89.9 (2)	C17—C12—C11	123.3 (7)
O3^i^—Ca1—O3	180.0	C13—C12—C11	106.1 (7)
O4^ii^—Ca2—O4	180.0 (3)	C14—C13—C12	108.8 (7)
O4^ii^—Ca2—O6	88.6 (2)	C14—C13—H13A	125.6
O4—Ca2—O6	91.4 (2)	C12—C13—H13A	125.6
O4^ii^—Ca2—O6^ii^	91.4 (2)	C13—C14—N3	108.4 (7)
O4—Ca2—O6^ii^	88.6 (2)	C13—C14—S1	130.8 (6)
O6—Ca2—O6^ii^	180.0	N3—C14—S1	120.7 (6)
O4^ii^—Ca2—O5^ii^	88.2 (2)	N3—C15—H15A	109.5
O4—Ca2—O5^ii^	91.8 (2)	N3—C15—H15B	109.5
O6—Ca2—O5^ii^	89.7 (2)	H15A—C15—H15B	109.5
O6^ii^—Ca2—O5^ii^	90.3 (2)	N3—C15—H15C	109.5
O4^ii^—Ca2—O5	91.8 (2)	H15A—C15—H15C	109.5
O4—Ca2—O5	88.2 (2)	H15B—C15—H15C	109.5
O6—Ca2—O5	90.3 (2)	O4—C16—N4	124.3 (7)
O6^ii^—Ca2—O5	89.7 (2)	O4—C16—C17	129.3 (7)
O5^ii^—Ca2—O5	180.0 (3)	N4—C16—C17	106.3 (7)
C1—O1—Ca1	152.9 (6)	C12—C17—C18	130.2 (7)
C6—O2—Ca1	137.1 (5)	C12—C17—C16	123.7 (7)
C11—O3—Ca1	151.9 (5)	C18—C17—C16	106.1 (7)
C16—O4—Ca2	162.2 (5)	C19—C18—C17	110.0 (7)
C21—O5—Ca2	149.4 (6)	C19—C18—H18A	125.0
C26—O6—Ca2	139.4 (6)	C17—C18—H18A	125.0
C1—N1—C5	121.3 (9)	C18—C19—N4	106.7 (7)
C1—N1—C4	117.0 (9)	C18—C19—S2	132.3 (6)
C5—N1—C4	121.6 (9)	N4—C19—S2	121.1 (6)
C6—N2—C10	125.4 (7)	N4—C20—H20A	109.5
C6—N2—C9	113.8 (7)	N4—C20—H20B	109.5
C10—N2—C9	120.6 (7)	H20A—C20—H20B	109.5
C11—N3—C14	110.1 (6)	N4—C20—H20C	109.5
C11—N3—C15	124.3 (6)	H20A—C20—H20C	109.5
C14—N3—C15	125.3 (7)	H20B—C20—H20C	109.5
C16—N4—C19	110.9 (7)	O5—C21—N5	125.2 (8)
C16—N4—C20	124.0 (6)	O5—C21—C22	124.8 (8)
C19—N4—C20	124.9 (7)	N5—C21—C22	109.9 (7)
C21—N5—C25	124.3 (8)	C21—C22—C23	102.2 (8)
C21—N5—C24	114.1 (7)	C21—C22—H22A	111.3
C25—N5—C24	121.5 (8)	C23—C22—H22A	111.3
C26—N6—C30	122.3 (9)	C21—C22—H22B	111.3
C26—N6—C29	116.1 (9)	C23—C22—H22B	111.3
C30—N6—C29	121.7 (8)	H22A—C22—H22B	109.2
O1—C1—N1	129.4 (10)	C24—C23—C22	104.4 (7)
O1—C1—C2	122.8 (9)	C24—C23—H23A	110.9
N1—C1—C2	107.8 (8)	C22—C23—H23A	110.9
C3—C2—C1	103.3 (8)	C24—C23—H23B	110.9
C3—C2—H2A	111.1	C22—C23—H23B	110.9
C1—C2—H2A	111.1	H23A—C23—H23B	108.9
C3—C2—H2B	111.1	N5—C24—C23	102.4 (7)
C1—C2—H2B	111.1	N5—C24—H24A	111.3
H2A—C2—H2B	109.1	C23—C24—H24A	111.3
C2—C3—C4	104.6 (8)	N5—C24—H24B	111.3
C2—C3—H3A	110.8	C23—C24—H24B	111.3
C4—C3—H3A	110.8	H24A—C24—H24B	109.2
C2—C3—H3B	110.8	N5—C25—H25A	109.5
C4—C3—H3B	110.8	N5—C25—H25B	109.5
H3A—C3—H3B	108.9	H25A—C25—H25B	109.5
N1—C4—C3	102.6 (7)	N5—C25—H25C	109.5
N1—C4—H4A	111.2	H25A—C25—H25C	109.5
C3—C4—H4A	111.2	H25B—C25—H25C	109.5
N1—C4—H4B	111.2	O6—C26—N6	128.3 (10)
C3—C4—H4B	111.2	O6—C26—C27	123.7 (8)
H4A—C4—H4B	109.2	N6—C26—C27	108.0 (8)
N1—C5—H5A	109.5	C28—C27—C26	102.0 (8)
N1—C5—H5B	109.5	C28—C27—H27A	111.4
H5A—C5—H5B	109.5	C26—C27—H27A	111.4
N1—C5—H5C	109.5	C28—C27—H27B	111.4
H5A—C5—H5C	109.5	C26—C27—H27B	111.4
H5B—C5—H5C	109.5	H27A—C27—H27B	109.2
O2—C6—N2	121.9 (8)	C27—C28—C29	106.0 (8)
O2—C6—C7	127.1 (7)	C27—C28—H28A	110.5
N2—C6—C7	111.0 (7)	C29—C28—H28A	110.5
C6—C7—C8	105.3 (7)	C27—C28—H28B	110.5
C6—C7—H7A	110.7	C29—C28—H28B	110.5
C8—C7—H7A	110.7	H28A—C28—H28B	108.7
C6—C7—H7B	110.7	N6—C29—C28	102.5 (8)
C8—C7—H7B	110.7	N6—C29—H29A	111.3
H7A—C7—H7B	108.8	C28—C29—H29A	111.3
C7—C8—C9	105.4 (7)	N6—C29—H29B	111.3
C7—C8—H8A	110.7	C28—C29—H29B	111.3
C9—C8—H8A	110.7	H29A—C29—H29B	109.2
C7—C8—H8B	110.7	N6—C30—H30A	109.5
C9—C8—H8B	110.7	N6—C30—H30B	109.5
H8A—C8—H8B	108.8	H30A—C30—H30B	109.5
N2—C9—C8	104.1 (7)	N6—C30—H30C	109.5
N2—C9—H9A	110.9	H30A—C30—H30C	109.5
C8—C9—H9A	110.9	H30B—C30—H30C	109.5
			
Ca1—O1—C1—N1	101.2 (16)	C19—N4—C16—O4	179.8 (7)
Ca1—O1—C1—C2	−77.4 (17)	C20—N4—C16—O4	3.3 (13)
C5—N1—C1—O1	2.4 (17)	C19—N4—C16—C17	−0.7 (9)
C4—N1—C1—O1	179.6 (10)	C20—N4—C16—C17	−177.2 (7)
C5—N1—C1—C2	−178.9 (9)	C13—C12—C17—C18	−179.8 (9)
C4—N1—C1—C2	−1.6 (12)	C11—C12—C17—C18	0.1 (14)
O1—C1—C2—C3	166.4 (9)	C13—C12—C17—C16	0.6 (14)
N1—C1—C2—C3	−12.5 (10)	C11—C12—C17—C16	−179.5 (7)
C1—C2—C3—C4	20.1 (10)	O4—C16—C17—C12	−0.6 (13)
C1—N1—C4—C3	14.5 (12)	N4—C16—C17—C12	180.0 (7)
C5—N1—C4—C3	−168.3 (9)	O4—C16—C17—C18	179.7 (8)
C2—C3—C4—N1	−20.9 (10)	N4—C16—C17—C18	0.3 (9)
Ca1—O2—C6—N2	148.7 (7)	C12—C17—C18—C19	−179.4 (8)
Ca1—O2—C6—C7	−32.1 (14)	C16—C17—C18—C19	0.3 (9)
C10—N2—C6—O2	−3.1 (13)	C17—C18—C19—N4	−0.7 (9)
C9—N2—C6—O2	−177.5 (8)	C17—C18—C19—S2	178.6 (7)
C10—N2—C6—C7	177.5 (8)	C16—N4—C19—C18	0.9 (9)
C9—N2—C6—C7	3.2 (10)	C20—N4—C19—C18	177.4 (7)
O2—C6—C7—C8	175.1 (9)	C16—N4—C19—S2	−178.5 (6)
N2—C6—C7—C8	−5.6 (10)	C20—N4—C19—S2	−2.1 (11)
C6—C7—C8—C9	5.7 (10)	Ca2—O5—C21—N5	−98.8 (13)
C6—N2—C9—C8	0.7 (10)	Ca2—O5—C21—C22	83.7 (15)
C10—N2—C9—C8	−174.0 (8)	C25—N5—C21—O5	0.7 (15)
C7—C8—C9—N2	−4.0 (10)	C24—N5—C21—O5	−177.7 (9)
Ca1—O3—C11—N3	53.7 (15)	C25—N5—C21—C22	178.5 (9)
Ca1—O3—C11—C12	−126.3 (10)	C24—N5—C21—C22	0.2 (11)
C14—N3—C11—O3	−179.7 (7)	O5—C21—C22—C23	−166.3 (9)
C15—N3—C11—O3	6.9 (12)	N5—C21—C22—C23	15.9 (10)
C14—N3—C11—C12	0.3 (8)	C21—C22—C23—C24	−24.7 (10)
C15—N3—C11—C12	−173.2 (7)	C21—N5—C24—C23	−16.2 (10)
O3—C11—C12—C17	−0.9 (14)	C25—N5—C24—C23	165.4 (9)
N3—C11—C12—C17	179.1 (7)	C22—C23—C24—N5	24.8 (10)
O3—C11—C12—C13	179.0 (8)	Ca2—O6—C26—N6	−154.4 (8)
N3—C11—C12—C13	−1.0 (9)	Ca2—O6—C26—C27	27.1 (14)
C17—C12—C13—C14	−178.8 (8)	C30—N6—C26—O6	0.6 (16)
C11—C12—C13—C14	1.3 (9)	C29—N6—C26—O6	179.3 (9)
C12—C13—C14—N3	−1.2 (10)	C30—N6—C26—C27	179.2 (9)
C12—C13—C14—S1	−177.1 (7)	C29—N6—C26—C27	−2.1 (11)
C11—N3—C14—C13	0.6 (9)	O6—C26—C27—C28	−165.5 (9)
C15—N3—C14—C13	173.9 (7)	N6—C26—C27—C28	15.8 (10)
C11—N3—C14—S1	177.0 (6)	C26—C27—C28—C29	−22.4 (10)
C15—N3—C14—S1	−9.7 (11)	C26—N6—C29—C28	−12.2 (12)
Ca2—O4—C16—N4	17 (2)	C30—N6—C29—C28	166.4 (9)
Ca2—O4—C16—C17	−161.9 (13)	C27—C28—C29—N6	21.6 (11)

Symmetry codes: (i) −*x*+1, −*y*, −*z*+1; (ii) −*x*+1, −*y*+1, −*z*.

### Hydrogen-bond geometry (Å, º)

**Table:**

*D*—H···*A*	*D*—H	H···*A*	*D*···*A*	*D*—H···*A*
C4—H4*A*···O3^iii^	0.99	2.51	3.456 (12)	159
C10—H10*B*···S2	0.98	2.97	3.820 (10)	146
C15—H15*A*···O2^i^	0.98	2.58	3.545 (10)	167
C20—H20*A*···O6	0.98	2.53	3.504 (11)	172
C22—H22*A*···S1^iv^	0.99	2.93	3.839 (11)	154
C23—H23*B*···N3^iv^	0.99	2.69	3.655 (12)	165
C27—H27*A*···O4^ii^	0.99	2.63	3.444 (11)	140

Symmetry codes: (i) −*x*+1, −*y*, −*z*+1; (ii) −*x*+1, −*y*+1, −*z*; (iii) −*x*, −*y*, −*z*+1; (iv) *x*, *y*+1, *z*.
